# Combinatorial experimental protocols for Erbicin-derived immunoagents and Herceptin

**DOI:** 10.1038/sj.bjc.6604022

**Published:** 2007-10-09

**Authors:** C De Lorenzo, F Troise, V Cafaro, G D'Alessio

**Affiliations:** 1 Dipartimento di Biologia Strutturale e Funzionale, Università di Napoli Federico II, via Cinthia, Napoli 80126, Italy

**Keywords:** immunotherapy, ErbB2, mammary carcinoma

## Abstract

Erbicin is a human anti-ErbB2 single-chain antibody fragment with high affinity and selectivity for ErbB2-positive cancer cells. Two anti-ErbB2 immunoconjugates, called Erb-hRNase and Erb-hcAb, have been prepared and found to be selectively cytotoxic on ErbB2-positive cancer cells *in vitro* and *vivo*. In Erb-hRNase, Erbicin is linked to a human RNase and in Erb-hcAb it is linked to the key structural and functional regions of a human IgG. Herceptin is an anti-ErbB2 humanised antibody successfully used in the immunotherapy of breast cancer. We report here that the Erbicin-derived immunoagents target on breast cancer cells an ErbB2 epitope different than that of Herceptin. This finding led us to verify the effects of Herceptin on breast cancer cells when it was used in combination with the Erbicin-derived immunoagents. The results indicated that in combination experiments the antitumour action of Herceptin and that of the novel agents were significantly increased in an additive fashion. An inspection of the mechanism of action of Erb-hRNase or Erb-hcAb combined with Herceptin provided evidence that the antibody combinations engendered an increased downregulation of the ErbB2 receptor, and led to an enhanced apoptotic cell death.

Breast cancer is the second most common female cancer worldwide. The major molecule implicated as a contributing cause for this malignancy is the transmembrane tyrosine kinase ErbB2 receptor. Its overexpression is a prognostic predictor of overall survival and time to relapse (Cooke *et al*, 2001). However, amplification of the *HER2/neu (ErbB2)* gene, and overexpression of the receptor occurs in only a fraction (up to 25%) of invasive breast cancers.

Herceptin, the only anti-ErbB2, humanised monoclonal antibody approved by FDA for therapy of mammary carcinoma is currently and successfully prescribed for treatment of ErbB2-positive breast cancer. However, in 40–60% of all patients with ErbB2-overexpressing tumours (Cardoso *et al*, 2002), Herceptin has little or no effect on tumour regression. For these patients, the prognosis is poor and the disease progresses more aggressively. To increase the response rate, combination treatments of Herceptin with anthracyclines have been performed, but unfortunately this leads to the onset of heart failure and cardiomyopathy. In fact, large-scale clinical studies with Herceptin have shown that up to 7% of patients suffer from cardiac disfunction when Herceptin is used in monotherapy, and 28% when it is combined with anthracyclines (Baselga, 2000; Slamon *et al*, 2001; Sparano, 2001). These facts indicate that novel immunotherapeutic strategies to fight ErbB2-positive carcinomas are strongly called for.

We have isolated Erbicin, a human anti-ErbB2 scFv (single-chain antibody fragment) (De Lorenzo *et al*, 2002), and used it to prepare two immunoconjugates. The first of these was a fully human anti-ErbB2 immunoRNase, called Erb-hRNase (De Lorenzo *et al*, 2004a), in which the toxin of a typical immunotoxin is replaced by a pro-toxin, a nontoxic human RNase. The RNase however becomes toxic when internalised in ErbB2-positive tumour cells, to which it is directed through its fusion with Erbicin. Another Erbicin-derived immunoconjugate was a human, compact antibody (De Lorenzo *et al*, 2004b) called Erb-hcAb. It was obtained by fusing human Erbicin to the CH2, CH3 and hinge regions from a human IgG1. Such antibody is described as ‘compact’ for its reduced size (100 kDa) when compared to that of a typical IgG (155 kDa).

Both immunoconjugates, Erb-hRNase and Erb-hcAb, were found to have a high affinity for ErbB2 with an apparent *K*_D_ in the nM range and selectively kill ErbB2-positive cells, both *in vitro* and *in vivo* (De Lorenzo *et al*, 2004a, 2004b, 2005), with no detectable effects on ErbB2-negative cells. The Erb-hcAb antibody has been found to possess, in contrast to Herceptin, not only antigen-dependent (ADCC) but also complement-dependent (CDC) cytotoxicity. Furthermore, its size, smaller than that of immunoglobulins, should favour penetration in solid tumours.

Here we show for the first time that these novel immunoagents recognise on the ErbB2 receptor an epitope different from that targeted by Herceptin. Based on this finding, we demonstrate that the novel anti-ErbB2 immunoagents can be successfully used in combination with Herceptin, and with conventional antineoplastic agents, such as taxol and *cis*-platin, to achieve more potent antitumour effects.

## MATERIALS AND METHODS

### Antibodies and cell cultures

The antibodies used were: Herceptin (Genentech, South San Francisco, CA, USA); HRP-conjugated anti-His antibody (Qiagen, Valencia, CA, USA); horseradish peroxidase-conjugated goat anti-human affinity isolated IgG (Fc specific) (Sigma, St Louis, MO, USA); horseradish peroxidase-conjugated rabbit anti-mouse immunoglobulin antibody (Pierce, Rockford, IL, USA). Erbicin, Erb-hcAb and Erb-hRNase were prepared as previously described (De Lorenzo *et al*, 2004a, 2004b).

SKBR3 cells (from ATCC, Rockville, MD, USA) were cultured in RPMI 1640 (Gibco BRL, Life Technologies, Paisley, UK) with 10% foetal bovine serum, 50 U ml^−1^ penicillin and 50 *μ*g ml^−1^ streptomycin (all from Gibco BRL). Hybridomas producing 7c2, MAB74 and 2c4 mAbs (ATCC) were grown in a 1 : 1 mixture of DMEM (Gibco) and RPMI 1640 supplemented with 10% heat-inactivated foetal bovine serum.

### Enzyme-linked immunosorbent assays

ErbB2-positive SKBR3 cells, harvested in nonenzymatic dissociation solution (Sigma), were washed and transferred to U-bottom microtitre plates (1 × 10^5^ cells per well). After blocking with PBS containing 6% bovine serum albumin (BSA), cells were treated with the antibodies in enzyme-linked immunosorbent assay (ELISA) buffer (PBS/BSA 3%) for 90 min. After centrifugation and removal of supernatants, the pelleted cells were washed twice in 200 *μ*l of ELISA buffer, resuspended in 100 *μ*l of ELISA buffer, and incubated with peroxidase-conjugated anti-His mAb (Qiagen) for scFv detection, with peroxidase-conjugated anti-human IgG (Fc-specific) antibody (Sigma) or peroxidase-conjugated anti-mouse IgG antibody (Pierce) for detection of human or murine antibodies, respectively. After 1 h, the plates were centrifuged, washed with ELISA buffer, and reacted with 3,3′,5,5′-tetramethylbenzidine (TMB) (Sigma). Binding values were determined from the absorbance at 450 nm, and reported as the mean of at least three determinations (standard deviation⩽5%).

### Cell morphology and apoptosis as induced by anti-ErbB2 antibodies

SKBR3 cells were plated at a density of 6 × 10^5^ well^−1^ in six-well plates and incubated at 37°C with either Herceptin (200 nM) or Erb-hcAb (200 nM) or with both antibodies simultaneously for 24–72 h. These concentrations were chosen to achieve maximal effects. After suitable time intervals, cells were harvested, washed in PBS, and treated with Annexin V-FITC following resuspension in binding buffer (10 mM HEPES, pH 7.4, 140 mM NaCl, 2.5 mM CaCl_2_). Labelled cells were analysed using the FACS Calibur flow cytometer (Becton Dickinson, Oxford, UK); the data were processed using CellQuest software (Becton Dickinson). The apoptotic inducer puromycin (10 *μ*g ml^−1^) was used as a positive control. After 72 h, cells from plates cultured in parallel were observed by light microscopy (Nikon ECLIPSE E1000, Melville, NY, USA), photographed (Nikon digital camera DXM 1200F), detached from the plates with cell dissociation solution (Sigma) and counted using the Trypan-Blue (Sigma) exclusion test.

### Cytotoxicity assays

ErbB2-positive cells were seeded in 96-well plates at a density of 1.5 × 10^4^ well^−1^ in 150 *μ*l. For the evaluation of the effects achieved by combining the chemotherapeutic drugs with the immunoagents, Taxol (Bristol-Myers Squibb, Sermoneta, Italy) or *cis*-platin (Sigma) were added at increasing concentrations in the presence or absence of Erb-hcAb (100 nM) or Erb-hRNase (10 nM).

For combinatorial treatment with Herceptin, given the lower cytotoxic effect of Herceptin, higher concentrations of Erbicin-derived immunoagents were used. Thus, cells were treated with Erb-hcAb (120 nM), Erb-hRNase (25 nM) or with a combination of each immunoagent with Herceptin (120 nM). After 72 h at 37°C, cell viability was determined in triplicate using the ATPlite 1 step test (Perkin Elmer, Cologno Monzese, Italy). The resulting luminescence was measured in a microplate counter (Multilabel Counter Victor 3, Perkin Elmer). Cell survival was expressed as percent of viable treated cells with respect to control untreated cultures. Typically, standard deviations were below 5%.

### Receptor downregulation assay

SKBR3 cells were plated at a density of 6 × 10^5^ well^−1^ in six-well plates. After 24 h, the medium was replaced by methionine-free medium containing 100 *μ*Ci of [^35^S]methionine ml^−1^ in 2% foetal bovine serum. Cells were metabolically labelled for 16 h and then incubated with Erb-hcAb or Herceptin (each at a concentration of 200 nM) or with both antibodies in fresh medium containing unlabelled methionine. After 16 h, whole-cell lysates were prepared and equal amounts, determined by radioactivity measurement, were subjected to immunoprecipitation with the C-terminal-specific anti-ErbB2 antibody Neu C-18 (Santa Cruz Biotechnology, Santa Cruz, CA, USA) and with an anti-actin mAb (Sigma). The immune complexes were collected by adsorption to protein A-Sepharose beads (Sigma) for 1 h at 4°C, then washed and released by boiling in electrophoresis loading buffer. Samples were separated on a 7.5% SDS–PAGE, and analysed by autoradiography. Band intensities were quantitated by using a scanner (Molecular Imager FX GS-710, Biorad, Hercules, CA, USA).

### Surface plasmon resonance analyses

Studies on the binding properties of Herceptin, Erb-hcAb and Erb-hRNase to the soluble ErbB2 receptor extracellular domain (ECD) were performed by surface plasmon resonance spectroscopy using a BIAcore X instrument (BiacoreAB, Uppsala, Sweden). Extracellular domain was immobilised to the surface of sensor chip CM5 (Biacore, Inc., Piscataway, NJ, USA) using standard amine coupling chemistry. During the coupling process, a constant flow rate of 10 *μ*l min^−1^ was maintained. The surface of the chip was activated with a 70 *μ*l mixture 1 : 1 of 0.4 M 1-ethyl-3-(3-dimethyl aminopropyl) carbodimide hydrochloride and 0.1 M *N*-hydroxysuccinimide (Biacore, Inc.), followed by 60 *μ*l of ECD (1 *μ*g ml^−1^) dissolved in 10 mM sodium acetate buffer, pH 4.5, to capture 500 RU of ECD. To block unreacted activated esters, 70 *μ*l of 1 M ethanolamine hydrochloride, pH 8.5, was finally injected, and the remaining noncovalently associated ECD was washed from the surface with four 30 s injections of 10 mM NaOH. The reference flow cell was inactivated by amine coupling chemistry, as already described without the ECD injection. The running buffer was HBS-EP buffer (10 mM HEPES with 0.15 M NaCl, 3.4 mM EDTA and 0.005% surfactant P20 at pH 7.4). The binding analyses of Erb-hcAb and Erb-hRNase to ECD immobilised on the surface were performed by two subsequent injections of 213 nM Erb-hcAb (corresponding to 426 nM binding site concentration) or 500 nM Erb-hRNase using a 2 min contact time for each injection at a constant flow rate of 5 *μ*l min^−1^. The concentrations of Herceptin, Erb-hcAb and Erb-hRNase were chosen after several experiments at different concentrations. For Herceptin, a saturating concentration was chosen; for the Erbicin-derived immunoagents, the minimum concentration that gave clear effects was chosen.

Sensor chip surface was regenerated by a 30 s injection of 25 mM NaOH/1 M NaCl. Competition in binding to ECD between Herceptin and Erb-hcAb or Erb-hRNase was investigated by saturating the sensor chip with Herceptin and injecting over the surface Erb-hcAb or Erb-hRNase. To this purpose, two subsequent injections of 440 nM Herceptin at a constant flow rate of 5 *μ*l min^−1^ were performed using 2 min contact time for each injection, followed by two subsequent injections of 213 nM Erb-hcAb or 500 nM Erb-hRNase using a 2 min contact time for each injection. No regeneration of sensor surface was made between injections.

## RESULTS

### The epitope recognised by Erbicin-derived immunoagents is different from that of Herceptin

The apparent binding affinity of the compact antibody (Erb-hcAb) for the ErbB2 receptor on SKBR3 cells, as measured with ELISA assays, has been found to be about 1 nM (De Lorenzo *et al*, 2004b). This value is close to the values of 4 and 5 nM reported in the same paper for the parental Erbicin and Herceptin, respectively.

To determine if the novel immunoagents recognise an epitope different from that targeted by Herceptin, competition experiments were carried out by repeating the ELISA assays in the presence of Herceptin.

**Figure 1:**
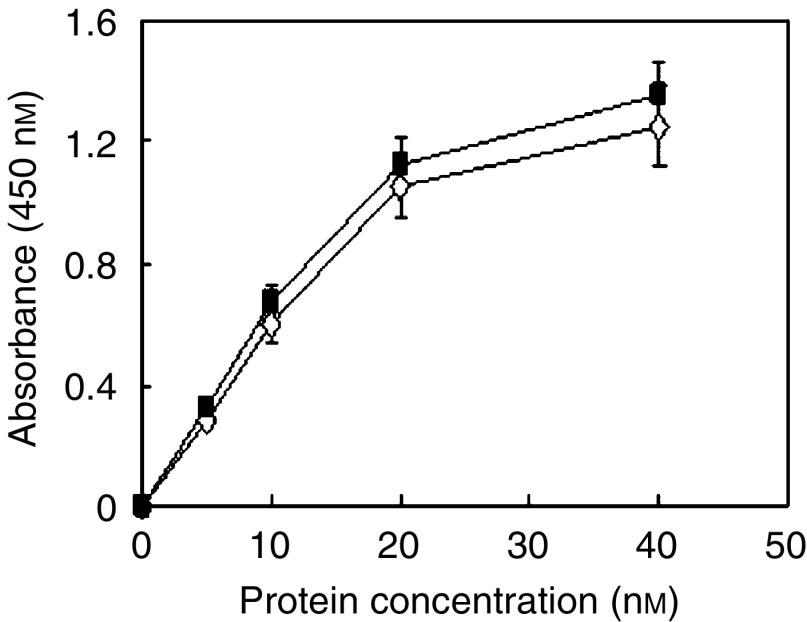
Binding curves of Erbicin to SKBR3 cells obtained through ELISA assays performed in the absence (empty rhomboids) or in the presence (black squares) of Herceptin.

In these experiments, the parental scFv (Erbicin) was added at increasing concentrations (5–40 nM) to ErbB2-positive cells preincubated with Herceptin at a saturating concentration (50 nM) for 1 h, or to untreated cells. Binding was detected with a peroxidase-conjugated anti-His mAb capable to reveal the scFv. As shown in Figure 1, the binding curves of Erbicin obtained in the absence or presence of Herceptin were found to be superimposable. These results strongly suggest that the epitope recognised by the scFv is different from that of Herceptin. This would clearly apply also to the immunoconjugates (Erb-hcAb and Erb-hRNase) in which Erbicin is the immunomoiety.

To test whether other available anti-ErbB2 monoclonal antibodies such as 2c4 (Pertuzumab precursor), 7c2 and MAB74 recognise different epitopes from that of Erbicin-derived immunoagents, binding assays were performed by adding each mAb preparation to SKBR3 cells previously incubated with Erb-hcAb at a saturating concentration (50 nM) for 1 h, or to untreated cells.

The binding ability of all three mAbs, detected with a peroxidase-conjugated anti-mouse secondary antibody (data not shown), was unaffected by the presence of Erb-hcAb. These results strongly suggest that the epitope recognised by the Erbicin-derived compact antibody is different from that bound by the three antibodies tested.

**Figure 2:**
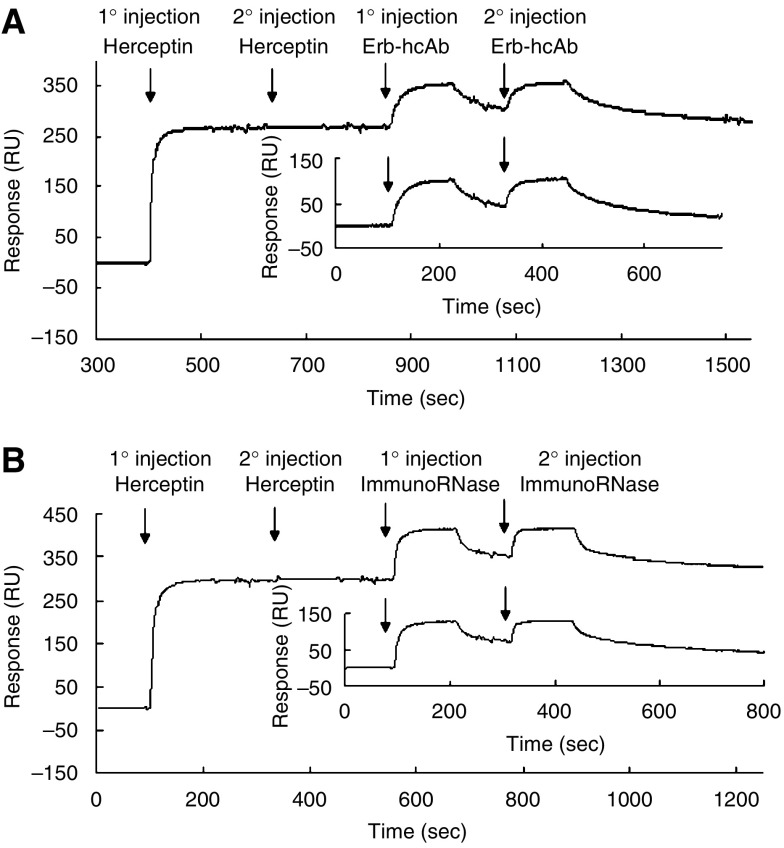
Binding of Erb-hcAb (**A**) and Erb-hRNase (**B**) to the ErbB2 extracellular domain (ECD) immobilised on plasmon resonance chips, before or after its saturation with Herceptin. From left to right: ECD saturated with Herceptin by two subsequent injections (marked by arrows) before the addition of either Erb-hcAb (**A**) or Erb-hRNase (**B**) by two subsequent injections (marked by arrows) over the chip. The insets show the binding of Erb-hcAb (**A**) or Erb-hRNase (**B**) to the ECD in the absence of Herceptin.

The competition, or lack of, between the Erb-hcAb and Erb-hRNase immunoagents and Herceptin for binding to the ErbB2 ECD was then tested with plasmon resonance experiments. Erb-hcAb and Erb-hRNase were each injected over ECD immobilised on the chip, before and after saturation with Herceptin (Figure 2).

After the ECD surface was saturated by repeated injections with Herceptin until no significant additional response was observed (Figure 2), equivalent amounts of each immunoagent were injected in parallel experiments. Both binding curves of Erb-hcAb and Erb-hRNase were found to be similar to those obtained in the absence of Herceptin (Figure 2, inset). This indicates that each immunoagent could bind to the ECD on the chip surface irrespective of the previous saturation with Herceptin.

It should be noted that in the case of Herceptin, no signs of dissociation were detectable. In the case instead of the Erbicin-derived immunoagents, the dissociation phase is already evident at about 120 s, as soon as the washings started. This is likely due to different *k*_off_ values. In fact, the dissociation phase, when no rebinding of analytes to the surface occurs, depends on the dissociation rate constant (*k*_off_), but it is independent on the association rate constant (*k*_on_).

In conclusion, both the results of the ELISA assays and those of the plasmon resonance assays show that the novel immunoagents do not compete with Herceptin for binding to ErbB2, and hence they bind on a site different from that of Herceptin.

### Effects on cell survival of Erbicin conjugates in combination with Herceptin

It has been reported (Harwerth *et al*, 1993; Hurwitz *et al*, 1995) that treatment with anti-ErbB2 antibodies that bind to different domains of the ErbB2 receptor leads to antitumour effects more potent than those observed when the cells are treated with each antibody alone.

On the basis of the observation that Herceptin and Erbicin-derived immunoagents bind to different epitopes on the receptor, we compared the growth inhibition effects of the compact antibody and the immunoRNase when administered alone or in combination with Herceptin.

**Figure 3:**
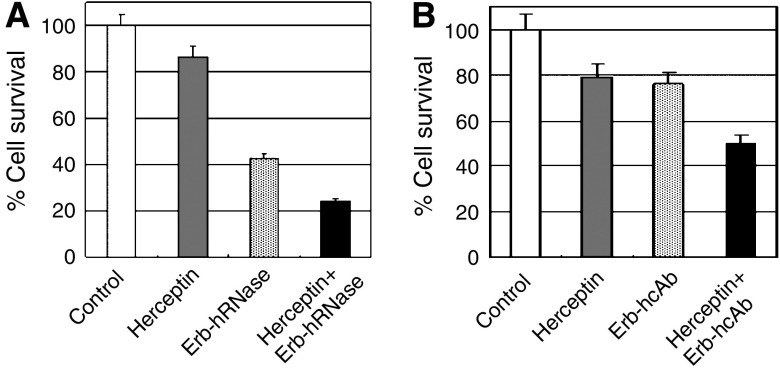
Growth inhibition effects on ErbB2-positive SKBR3 cells of combinatorial treatments of Erb-hRNase (**A**) or Erb-hcAb (**B**) with Herceptin.

ErbB2-positive cells were treated for 72 h at 37°C with Herceptin (120 nM), Erb-hcAb (120 nM), Erb-hRNase (25 nM) or with a combination of each immunoagent with Herceptin at the same concentrations. As shown in Figure 3, the combinatorial treatment inhibited the growth of SKBR3 cells more effectively than the treatment with each single immunoagent, thus showing additive interactions.

**Figure 4:**
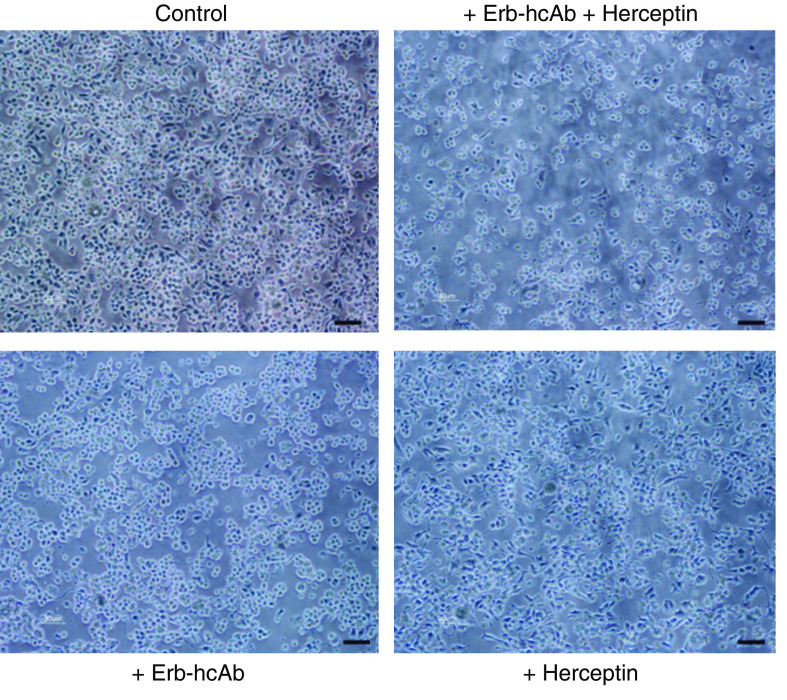
Effects on cell morphology of SKBR3 cells grown for 3 days in the absence (control) or in the presence of Herceptin, Erb-hcAb or both antibodies. Bar, 30 *μ*m.

Figure 4 shows the morphologic changes of SKBR3 cells after treatment for 72 h at 37°C with Herceptin (200 nM), Erb-hcAb (200 nM) or with the combination of the two immunoagents at the same concentrations. The combinatorial treatment clearly caused an increased cell death than each antibody alone, and more marked changes in cell morphology. Cells appeared to loose their typical epithelial features and assume distorted, round-shaped forms.

### Effects of combination of Erb-hcAb and Herceptin on ErbB2 levels

One possible mechanism underlying the antiproliferative effects of anti-ErbB2 antibodies is the downregulation of the receptor. Previous studies have shown that the effect of the Erbicin-derived compact antibody on tumour cell growth correlates with the lower ErbB2 levels generated by an increased receptor turnover as triggered by antibody binding (De Lorenzo *et al*, 2005). On the other hand, the high cytotoxicity of some anti-ErbB2 antibodies has been correlated with an increased degradation of ErbB2 (Hurwitz *et al*, 1995; Klapper *et al*, 1997).

**Figure 5:**
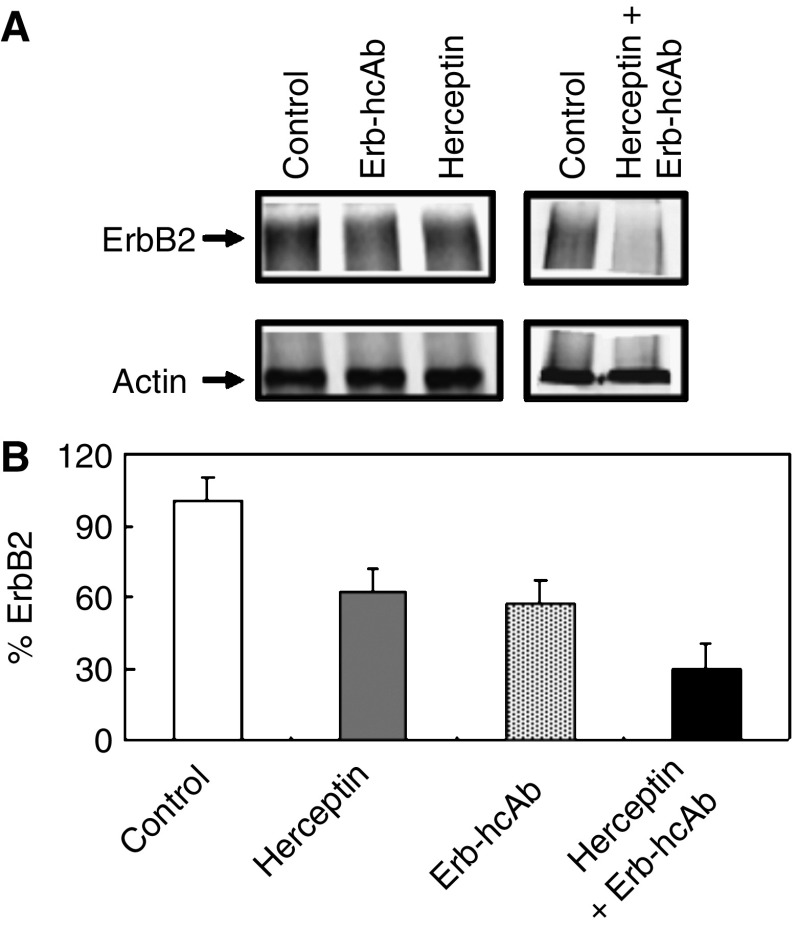
Turnover of ErbB2 in SKBR3 cells treated with anti-ErbB2 antibodies. (**A**) Autoradiograms of immunoprecipitated radioactive ErbB2 and actin (for normalization) from lysates of SKBR3 cells untreated or treated as indicated for 24 h with Erb-hcAb, Herceptin or with both antibodies simultaneously. Equal amounts (counts per minute) of ^35^S-labelled lysates were used. (**B**) The levels of ErbB2 are reported as percentages of the level detected in untreated control cells.

To test the effects on receptor downregulation of treatment with combined antibodies, SKBR3 cells were metabolically labelled, then treated for 16 h with Erb-hcAb (200 nM), or Herceptin (200 nM) or the combination of the two antibodies at the same concentration. These concentrations were chosen to achieve the degradation of the receptor, as previously reported (De Lorenzo *et al*, 2005). The receptor was immunoprecipitated with an anti-ErbB2 mAb from aliquots of cell lysates containing equal amounts of radioactivity, and analysed by SDS–PAGE and autoradiography. The results of the experiments, shown in Figure 5, indicated a more severe reduction of the ErbB2 level when the immunoagents were used in combination.

Densitometric evaluation of the data consistently indicated that the level of ErbB2 was reduced by 40% after exposure of SKBR3 cells to either Herceptin or Erb-hcAb alone, and by more than 70% when the two antibodies were used in combination. These results suggest that both antibodies can effectively induce the downregulation of the receptor, but the combinatorial treatment is more effective than the treatment with each single antibody.

### Determination of apoptosis

To determine whether the combination of Erb-hcAb and Herceptin induced a higher extent of apoptosis, we used annexin V to measure the appearance of phosphatidylserine, a marker of apoptosis, on the outer leaflet of the plasma membrane of SKBR3 cells. Cells were treated for 24 h with Erb-hcAb, or Herceptin or a combination of the two antibodies. The combinatorial treatment induced a two-fold increase in the percentage of apoptotic cells than that observed with each single antibody treatment. We found 25% of positive cells when the combined agents were used, and 10 and 14%, respectively, when Herceptin and Erb-hcAb were used alone. This indicated that Erb-hcAb and Herceptin have additive effects on growth inhibition through apoptosis.

### Effects on cell survival of Erbicin-derived immunoagents in combination with taxol or *cis*-platin

Previous reports (Ciardiello *et al*, 2000) have demonstrated that ErbB2 overexpression confers to malignant cells chemoresistance to anticancer drugs, as it activates the cycline-dependent kinase inhibitor p21^Cip1^, which in turn inhibits the p34^cdc2^ kinase (Yu *et al*, 1998). Thus, the activation of the latter kinase is required for chemotherapy-induced apoptosis, and its inhibition can be circumvented by antibody-mediated downregulation of the receptor. On the basis of these observations, we investigated if downregulation of ErbB2 due to the action of the human anti-ErbB2 Erb-hcAb could restore sensitivity to chemotherapy-induced apoptosis. This would lead to a superior antitumour efficacy of the chemotherapeutic drugs, when given in combination with Erb-hcAb.

**Figure 6:**
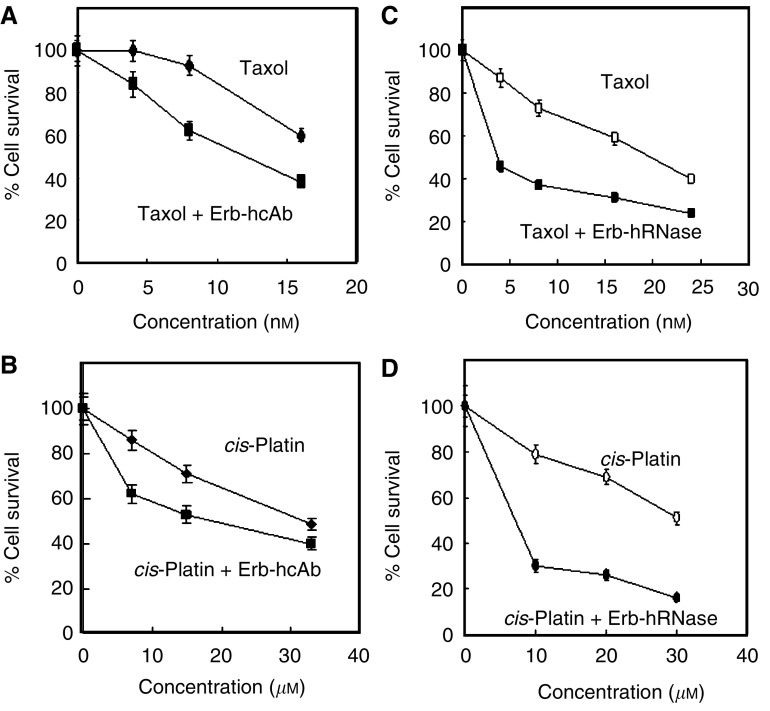
*In vitro* combined treatment of Erb-hcAb and Erb-hRNase with taxol or *cis*-platin. Left: Dose–response curves of SKBR3 cells treated for 72 h with taxol (**A**) or *cis*-platin (**B**) in the absence (rhomboids) or in the presence of Erb-hcAb (squares). The concentration of Erb-hcAb was kept constant at 100 nM. Cell survival in the control experiment performed by using Erb-hcAb as a single agent was at 82%. Right: Dose–response curves of SKBR3 cells treated for 72 h with taxol (**C**) or with *cis*-platin (**D**) in the absence (empty symbols) or presence of Erb-hRNase (black symbols). The concentration of Erb-hRNase was kept constant at 10 nM. Cell survival in the control experiment performed by using Erb-hRNase as a single agent was at 67%.

Taxol and *cis*-platin are chemotherapeutic agents which have already been shown to exert additive effects when used in combination with Herceptin (Baselga *et al*, 1998). We thus performed experiments to evaluate the effects of the combination of Erb-hcAb with taxol or *cis*-platin. Cytotoxicity assays were carried out on SKBR3 ErbB2-positive cells treated for 72 h at 37°C with either taxol (8–16 nM) or *cis*-platin (10–35 *μ*M) or Erb-hcAb (100 nM) or with a combination of each drug and Erb-hcAb at the same concentration. We found that the antitumour efficacy was clearly superior when the chemotherapeutic drugs were given in combination with Erb-hcAb (Figure 6A and B).

Similar experiments were performed with the chimaeric Erb-hRNase. SKBR3 cells were treated for 72 h at 37°C with either taxol (4–24 nM) or *cis*-platin (10–30 *μ*M) or Erb-hRNase (10 nM) alone or with a combination of each drug with Erb-hRNase at the same concentration. As shown in Figure 6C and D, both taxol and *cis*-platin displayed a much stronger cytotoxic effect when used in combination with Erb-hRNase.

The results strongly indicate that the combination of either taxol or *cis*-platin with the Erbicin-derived immunoagents produced additive effects, yielding synergistic interaction only when *cis*-platin was used in combination with Erb-hRNase.

## DISCUSSION

In the last few years, we have prepared and characterised new human anti-ErbB2 immunoagents based on Erbicin, an scFv with high affinity and selective cytotoxicity for ErbB2-positive cells (De Lorenzo *et al*, 2002). They are, as described in Introduction: (1) Erb-hRNase, an immuno-pro-toxin that contains a nontoxic human RNase, which becomes toxic when tethered by the Erbicin moiety inside tumour cells (De Lorenzo *et al*, 2004a); (2) Erb-hcAb, a compact, reduced-size anti-ErbB2 antibody engineered with Erbicin and the essential parts of a human immunoglobulin (De Lorenzo *et al*, 2004b).

A rational approach to the evaluation of these new anti-ErbB2 immunoagents as potential antitumour agents is the comparison of their properties with those of Herceptin (trastuzumab), now established as a powerful therapeutic tool for ErbB2-overexpressing breast cancer, and other carcinomas (Stebbing *et al*, 2000; Scholl *et al*, 2001). As Herceptin has antitumour activity in only one out of three of the patients, it is ineffective on ErbB2-low-expressing tumour cells, and patients often develop resistance to the drug after about one year of treatment, the attention has been directed towards alternative strategies, such as the use of combinatorial therapeutic protocols. Herceptin effectiveness has been found to improve when administered in combination with chemotherapeutic drugs, such as paclitaxel (Slamon *et al*, 2001; Merlin *et al*, 2002) or other anti-ErbB2 antibodies (Spiridon *et al*, 2002; Willems *et al*, 2005). As for the latter approach, it is necessary that the antibodies used in combination with Herceptin are directed to epitopes on the target cells different from that recognised by Herceptin.

We report here that the growth inhibition effects exerted by Erb-hRNase and Erb-hcAb on ErbB2-positive cells are significantly increased when used in combination with either chemotherapeutic drugs, or Herceptin. The effectiveness of taxol and *cis*-platin was found to increase more than two-fold when these drugs were used in combination with either Erb-hRNase or Erb-hcAb. Likewise, the cytotoxic action of Herceptin was enhanced by two- to three-fold when ErbB2-positive cells were treated with Herceptin combined with Erb-hRNase or Erb-hcAb.

The results obtained with Herceptin combined with the Erbicin-based immunoagents can be explained by the finding, reported here for the first time, that the epitope recognised by the Erbicin-based immunoagents is different from that targeted by Herceptin. This observation is supported by results from two different types of binding tests: ELISA assays and plasmon resonance analyses.

The ability of the Erbicin-derived immunoagents to target an ErbB2 epitope different from that targeted by Herceptin may also explain recent, preliminary results from our laboratory (De Lorenzo *et al*, unpublished), which suggest that these immunoagents have no cardiotoxicity effects on treated cells, as Herceptin does (Chien, 2000; Sparano, 2001), and appear to be cytotoxic also on Herceptin-resistant cells (De Lorenzo *et al*, manuscript submitted).

The investigated mechanistic features of the combined action of Erb-hRNase or Erb-hcAb with Herceptin have revealed a highly enhanced downregulation of the ErbB2 receptor, and clear signs of apoptotic toxicity.

In conclusion, the results reported here indicate that Erbicin-derived immunoagents have promising antitumour potential and can be favourably used in combination with Herceptin and/or conventional chemotherapeutic drugs.
